# Insanity and the Neuropathic Inheritance

**Published:** 1913-02-08

**Authors:** F. W. Mott

**Affiliations:** Pathologist, London County Mental Hospitals.


### INSANITY AND THE NEUROPATHIC INHERITANCE.

### By Dr. F. W. Mott, F.R.S., Pathologist, London County Mental Hospitals.

### Introductory.

On Tuesday, January 28, before the Royal Society of Medicine, Dr. F. W. Mott, F.R.S., read a paper before the Section of Psychiatry on "Insanity and the Neuropathic Inheritance," Sir George Savage presiding.

Dr. Mott said every case of insanity should be regarded as a biological problem, and the study resolved itself into the acquirement of a knowledge of what the individual was born with—Nature—and what had happened after birth—nurture. The former of these could be ascertained by a study of the ancestral stocks. The neuropathic tendency might be manifested in different members of the stock in different ways. Just as bodily features were transmitted from one generation to another, so was temperament.

Special racial and family characters were of later development, therefore were far less fixed, stable, and organised in the nervous system. Galton found that twins developed from one ovum and therefore identical germ plasm, living in a different environment, remained similar in temperament and character, whilst dissimilar twins, brought up and living in the same environment remained dissimilar. Galton also made a statistical inquiry into the inheritance of good and bad tempers, and concluded that one set of influences tended to mix good and bad at haphazard, another tended to assimilate them, another tended to divide families into contracted portions; and in that way there was a tendency to revert to the normal average of the race. Morel had pointed out that nervous irritable weakness and neurasthenic predisposition might be the first evidence of degeneration of a stock. The signs of degeneracy might be manifested as self-centred narrow-mindedness in religious beliefs, fanaticism, mysticism, spiritism, and unwholesome contempt of traditional custom, social usages, and morality, a vain spirit of spurious art and culture, a false, self-loving vanity in the pursuit of a sentimental altruism, the intelligence being well preserved. Such qualities might be combined with latent or active genius; but the brilliant intellectual qualities of a degenerate were generally associated with either a lack of moral sense or of sound judgment and higher control.

### The Madmen of History.

Dr. Mott declared that the world's history had been made by men who were either epileptics, insane, or born of a neuropathic stock: Alexander the Great, Julius Cæsar, Napoleon, Peter the Great, Frederick the Great, Pitt, Earl of Chatham, Mahomet, the Apostle Paul, Martin Luther, Emanuel Swedenborg, and others. Among English poets, Cowper, Wordsworth, Byron, Gray, Burns, Chatterton, Thomson were either insane or possessed the neuropathic temperament.

### Heredity in Relation to Insanity—Facts from London County Mental Hospitals.

Dr. Mott then proceeded to describe the work which had been done in the pathological laboratories of the London County Mental Hospitals on heredity in relation to insanity, chiefly by means of a card system which he instituted four years ago, the cases inquired into having reached 3,118, concerning 1,450 families. That showed the following facts: (1) In the insane offspring of insane parents, daughters were much more numerous than sons. (2) Amongst the insane members of the same family (brothers and sisters), sisters were more numerous than brothers, which fact might be usefully correlated with the further fact that there were more women in asylums than men. More than half the residents of the London County Mental Hospitals had been resident there more than ten years. Among the reasons for the preponderance of women inmates were that general paralysis, which was three times more frequent in men than in women, was a fatal disease; again, women were more likely to be insane because of the physiological emergencies connected with reproduction; moreover, women had a more unstable nervous system or mental equilibrium. To those factors must be added, in his opinion, the enforced suppression, by modern social conditions, of the reproductive functions and the maternal instinct in women of emotional temperament. The author then proceeded to establish the principle of anticipation or antedating, and Nature's means of mending or ending degenerate stock, showing a number of pedigrees, one of which exhibited the elimination of unsound elements in a stock in which there was a dual insane inheritance. It was generally admitted that in the pedigrees of general paralysis of the insane the neuropathic taint was not found to anything like the extent that it was in the pedigrees of patients suffering from neuroses, psychoses, and feeble-mindedness. This was not surprising, seeing that general paralysis was an organic disease due to the action of the syphilitic organism. He had been able to support his contentions by the investigation of upwards of 3,000 relatives of persons admitted into the London County Mental Hospitals.

